# Development and validation of deep learning-based automatic brain segmentation for East Asians: A comparison with Freesurfer

**DOI:** 10.3389/fnins.2023.1157738

**Published:** 2023-05-12

**Authors:** Chung-Man Moon, Yun Young Lee, Ki-Eun Hyeong, Woong Yoon, Byung Hyun Baek, Suk-Hee Heo, Sang-Soo Shin, Seul Kee Kim

**Affiliations:** ^1^Research Institute of Medical Sciences, Chonnam National University, Gwangju, Republic of Korea; ^2^Department of Radiology, Chonnam National University Hospital, Gwangju, Republic of Korea; ^3^Neurozen Inc., Seoul, Republic of Korea; ^4^Department of Radiology, Chonnam National University Medical School, Gwangju, Republic of Korea; ^5^Department of Radiology, Chonnam National University Hwasun Hospital, Hwasun, Republic of Korea

**Keywords:** brain volumetry, deep learning, magnetic resonance imaging, segmentation, ground truth

## Abstract

**Purpose:**

To develop and validate deep learning-based automatic brain segmentation for East Asians with comparison to data for healthy controls from Freesurfer based on a ground truth.

**Methods:**

A total of 30 healthy participants were enrolled and underwent T1-weighted magnetic resonance imaging (MRI) using a 3-tesla MRI system. Our Neuro I software was developed based on a three-dimensional convolutional neural networks (CNNs)-based, deep-learning algorithm, which was trained using data for 776 healthy Koreans with normal cognition. Dice coefficient (D) was calculated for each brain segment and compared with control data by paired *t*-test. The inter-method reliability was assessed by intraclass correlation coefficient (ICC) and effect size. Pearson correlation analysis was applied to assess the relationship between D values for each method and participant ages.

**Results:**

The D values obtained from Freesurfer (ver6.0) were significantly lower than those from Neuro I. The histogram of the Freesurfer results showed remarkable differences in the distribution of D values from Neuro I. Overall, D values obtained by Freesurfer and Neuro I showed positive correlations, but the slopes and intercepts were significantly different. It was showed the largest effect sizes ranged 1.07–3.22, and ICC also showed significantly poor to moderate correlations between the two methods (0.498 ≤ ICC ≤ 0.688). For Neuro I, D values resulted in reduced residuals when fitting data to a line of best fit, and indicated consistent values corresponding to each age, even in young and older adults.

**Conclusion:**

Freesurfer and Neuro I were not equivalent when compared to a ground truth, where Neuro I exhibited higher performance. We suggest that Neuro I is a useful alternative for the assessment of the brain volume.

## 1. Introduction

Quantitative regional brain volumetry in humans is of great importance in clinical practice for evaluating various neurologic diseases (Heo et al., 2022), and developmental or behavioral conditions arising from normal aging (Zhao et al., 2019). Previous brain magnetic resonance imaging (MRI) studies (Zhao et al., 2019; Heo et al., 2022) have been widely applied to quantify the volume, thickness, and other morphometrics of specific brain structures. In MRI-based volumetry methods, accurate brain segmentation with a short data-processing period, such as 5-10 min, is necessary to obtain precise quantitative values of brain volume and cortical thickness, especially in large datasets (Tustison et al., 2014).

In volumetric neuroimaging studies, segmentation of brain anatomy has been a key image- processing step (Srinivasan et al., 2020). Traditionally, manual segmentation was considered the gold standard approach for brain tissue measurement (Morey et al., 2009; Schoemaker et al., 2016); however this method is subjective, extremely time-consuming, laborious, and human-resource intensive, and thus unfeasible for large MRI datasets (Perlaki et al., 2017). Currently available algorithms have low clinical feasibility because of the long processing time for brain segmentation (Suh et al., 2020). Also, the major practical limitation of prior studies is incomplete segmentation of the brain into finer anatomic regions when using widely available tools such as Freesurfer (Kaku et al., 2019). Indeed, there are substantial challenges regarding how to obtain accurate segmentation of finer brain regions in a small brain size (Kaku et al., 2019). As such, automatic segmentation algorithms and software packages developed to label parts of brain MR images could drastically reduce processing time, enabling the analysis of large amounts of data, and could remove potential sources of inconsistency between sites (Velasco-Annis et al., 2018; Srinivasan et al., 2020). Recently, deep learning techniques, including convolutional neural networks (CNNs), have been employed predominantly for rapid and accurate segmentation of coarse regions of interest (ROIs) in the analysis of medical imaging data, and for reducing the long-term performance of computation (Bae et al., 2020; Thyreau and Taki, 2020). Another issue raised is that it may not be directly applicable to brain segmentation in East Asian individuals regarding data processing time and accuracy, because deep learning models were generally based on Caucasian brains.

Our clinical volumetry software program, Neuro I (Neurozen Inc., Seoul, Republic of Korea), was recently introduced to the neuroscience community; this program uses a three-dimensional (3D) CNN-based deep learning algorithm and is approved by the Food and Drug Administration (FDA) of Republic of Korea. Especially in this tool, 3D CNN-based deep learning algorithm was trained using 776 healthy Korean individuals with normal cognition to focus on the East Asian brain; which can generate 109 ROIs based on the Desikan-Killiany-Tourville (DKT) atlas. Unlike other clinical volumetry software, Neuro I also uses a deep learning segmentation module to increase accuracy of brain tissue extraction from non-brain structures, and to improve classification of brain tissues as white matter parcellation without manual correction. To our knowledge, evidence for the effect of deep learning automatic brain segmentation based on T1-weighted brain MR images using data from East Asians is limited. Also, there have been several methodological studies looking at the effects of different image segmentation strategies by comparing differences between software packages, but there were few studies with a manual gold standard considered as a ground truth (Grimm et al., 2015).

Here, we provide an exemplary comparison of Neuro I with Freesurfer (version 6.0), which is one of the most widely used automated segmentation methods among existing freely available tools (Pemberton et al., 2021). We hypothesized that the two different software packages use different segmentation procedures and are likely to produce different values.

Therefore, in this study, we aimed to compare subcortical volume measurements from the two software packages with a ground truth in healthy individuals, and to evaluate the inter-method reliability and correlation with ages.

## 2. Materials and methods

### 2.1. Study population

This study received Institutional Review Board approval, and the requirement for informed consent was waived due to the retrospective nature of the study. We searched the imaging database for 65 healthy individuals who underwent brain MRI at a university hospital between June 1, 2020 and November 30, 2021. The inclusion criterion for healthy controls was no clinical evidence of neurological or psychiatric symptoms, as evaluated by a physician. In total, 30 healthy individuals were included: 12 males and 18 females; age range, 30–77 years; mean age, 53.62 ± 13.52 years; bodyweight 60.58 ± 10.08 kg; and height 162.45 ± 8.33 cm.

### 2.2. MRI

All participants were scanned with a 3-tesla MRI scanner (Siemens, Erlangen, Germany) with a 12-channel head coil. High-resolution T1-weighted images were acquired using the MPRAGE sequence with the following parameters: repetition time/echo time = 2,530/3.37 ms; field-of-view = 256 mm × 256 mm; matrix = 256 × 256; and slice thickness = 1 mm. All MRI images were visually inspected by an experienced neuroradiologist to confirm appropriate image quality and to exclude individuals with visible brain abnormalities.

### 2.3. Magnetic resonance volumetry

Each volumetric T1-weighted image was used separately for analysis with two different software packages on a conventional desktop computer. For concision, not all brain structures were analyzed in this study. Both software packages provided volumes for the left and right hippocampus, amygdala, entorhinal cortex, inferior temporal gyrus, and middle temporal gyrus, which is not only of interest because its volume change reflects physiologic processes but it might also gain clinical significance as a neuroimaging biomarker for the main cognitive impairment and prognostic evaluation of Alzheimer’s disease (AD) (Onitsuka et al., 2004; Grimm et al., 2015; Zhou et al., 2016). Each of the regional volume measures was averaged across left and right hemispheres.

#### 2.3.1. Manual segmentation

To generate the ground truth for evaluating segmentation using ITK-SNAP version 3.8.0, a neuroradiologist with 20 years of dedicated experience in human brain MRI data processing and segmentation manually traced the all ROIs for the 30 participants; the scans had sufficient quality to allow manual tracing on gapless coronal slices following a detailed anatomic tracing protocol such as the DKT labeling protocol introduced in the Mindboggle publication (Klein and Tourville, 2012).

#### 2.3.2. Volumetric procedures

FreeSurfer 6.0 (Harvard University, Boston, MA, USA) was used to analyze structural MRI data according to procedures described in prior publications (Dale et al., 1999). Data were post-processed using the “recon-all” script to produce fully segmented regions. The processing requires approximately 8 h per 3D T1-weighted image. The volume measures of the ROIs were derived from the standard stats directory using the Desikan atlas.

**FIGURE 1:**
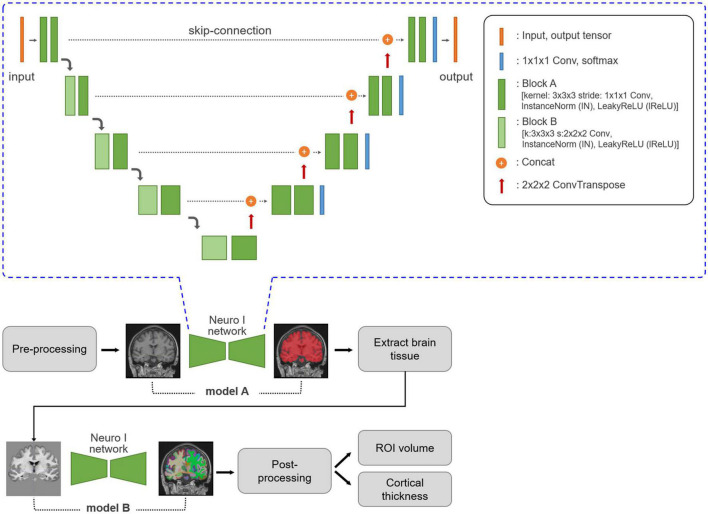
Neuro I process and network architecture. Three-dimensional T1-weighted MR images are subjected to MRI pre-processing, including registration and normalization. Based on empiric results showing good performance in our dataset, the Neuro I pipeline is constructed with two separate models. The first model, A, is a model for brain tissue segmentation. Using a mask from this model, sequentially after removing the intracranial volume, brain tissue is extracted from the original T1-weighted MR image to focus on classifying ROI types in brain tissue by removing non-targeted voxel classes. Model B extracts 109 brain ROIs by performing parcellation. Then, the volume and cortical thickness (mm) for each ROI are calculated. The Neuro I network is the common network structure of the two deep learning models included in the Neuro I software. The two models differ only in the shape of the input and output tensors. The above architecture is the same as that of nnU-Net (Isensee et al., 2021), but some hyperparameters and the learning process are customized because nnU-Net pipeline should be adapted to our model environment such as patch size, and GPU memory available when inputting brain MRI datasets.

Neuro I used a deep learning algorithm applied to multiple steps, such as analysis-failure prediction, intensity normalization, brain extraction, and segmentation. Values for the volumes of regional brain structures and cortical thickness were obtained (Figure 1). All processing was completed within 10 min.

### 2.4. Statistical analyses

Spatial overlap (Dice coefficient, D) for each ROI was calculated between the different segmentations and a ground truth according to the following equations:


$$D=2\times \frac{A\cap B}{A+B}$$

where A is the segmented voxels by two different methods, B is the voxels of a ground truth, and ∩ is the intersection operation. The maximal value of D is 1, indicating perfect overlap between the two segmentations, while decreasing D indicates less overlap.

Data were analyzed using SPSS 20.0 software (SPSS Inc., Chicago, IL, USA) as a statistical analysis tool to compare the D values resulting from different segmentation approaches by paired *t*-test. Moreover, histograms of the distribution of D values were computed. Inter-method reliability was assessed by calculating the intraclass correlation coefficient (ICC) and effect size obtained from the standardized mean difference between the D values from the two methods. The guidelines used to interpret effect size (Cohen’s d) values were as follows: small, *d* = 0.2; medium, *d* = 0.5; and large, *d* = 0.8 (Cohen, 1988). Pearson correlation analysis was used to analyze the agreement of D values between the two methods, and between D values and ages for each method. The significance level was set at *p* < 0.05 for all the analyses.

### 2.5. Methodological considerations

We used manual segmentation as a ground truth because this is commonly used as the reference technique for assessing the performance of automatic segmentation techniques, and manual tracing represents the true boundaries of the segmented structures (Perlaki et al., 2017). To calculate D values, Freesurfer and ground truth segmentations had to be transformed from the image space of Freesurfer and a ground truth back to Neuro I space, which may result in slight alterations due to resampling (Morey et al., 2009; Dewey et al., 2010).

## 3. Results

### 3.1. Segmentation

Magnetic resonance imaging scans for the 30 participants were segmented twice each, once by each algorithm. For each scan, total computational time was approximately 8 h for Freesurfer, and 10 min for Neuro I. Table 1 presents mean volume measurements, as delineated by each method, including the ground truth. Mean intra-subject standard deviations are also reported.

**TABLE 1:** Average structure volumes of Freesurfer, Neuro I, and ground truth.

	Freesurfer		Neuro I		Ground truth	
**Region**	**mL**	**(SD)**	**mL**	**(SD)**	**mL**	**(SD)**
Hippocampus	3.906	(0.389)	4.124	(0.458)	3.642	(0.453)
Amygdala	1.497	(0.218)	1.568	(0.254)	1.486	(0.224)
Entorhinal cortex	1.738	(0.288)	1.184	(0.248)	1.138	(0.228)
Inferior temporal gyrus	11.767	(1.615)	11.985	(1.543)	11.121	(1.454)
Middle temporal gyrus	13.298	(1.741)	13.785	(1.883)	12.215	(1.552)

Mean volumes shown with mean intra-subject standard deviation in parentheses. mL, milliliter.

**FIGURE 2:**
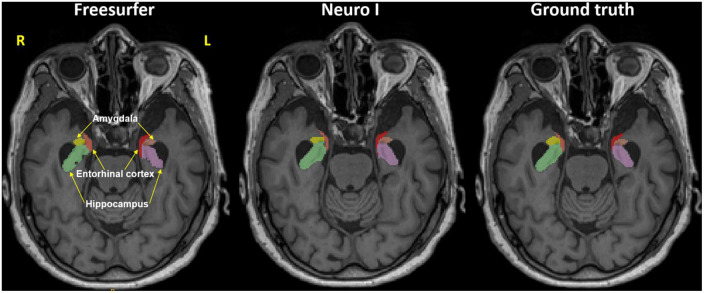
Representative MR image in the axial plane showing the hippocampus, amygdala, and entorhinal cortex in Freesurfer **(left)**, our proposed model (Neuro I) **(middle)**, and ground truth **(right)**. It is evident that Freesurfer has errors in both over and underestimation along the boundaries of brain regions, as well as non-natural looking with grainy segmentation, whereas Neuro I segmentation obeys well the segment boundaries with more natural looking, rendering a smooth contoured. R, right; L, left.

Regarding errors of segmentation in the hippocampus, amygdala, and entorhinal cortex, Freesurfer segmentation seemed to necessitate manual corrections for quality control (Klapwijk et al., 2019), showing major boundary errors, such as stair-step artifacts along the boundary or non-natural looking, relative to Neuro I and ground truth, as demonstrated in Figure 2.

### 3.2. Comparison of dice coefficients for the different segmentation methods

**FIGURE 3:**
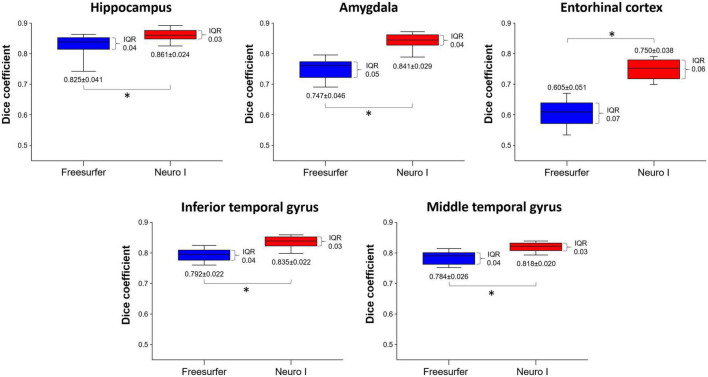
Comparison of dice coefficients obtained from Freesurfer and Neuro I using paired *t*-test. IQR, interquartile range. *Statistically significant difference at *p* < 0.05.

Figure 3 shows the effects of the different segmentation methods on D values. The mean D values of all ROIs obtained after processing with Freesurfer were significantly lower than those values obtained with Neuro I (paired *t*-test, *p* < 0.001). Also, the D values processed by Freesurfer had a larger spread [interquartile range (IQR)] than values processed by Neuro I.

**FIGURE 4:**
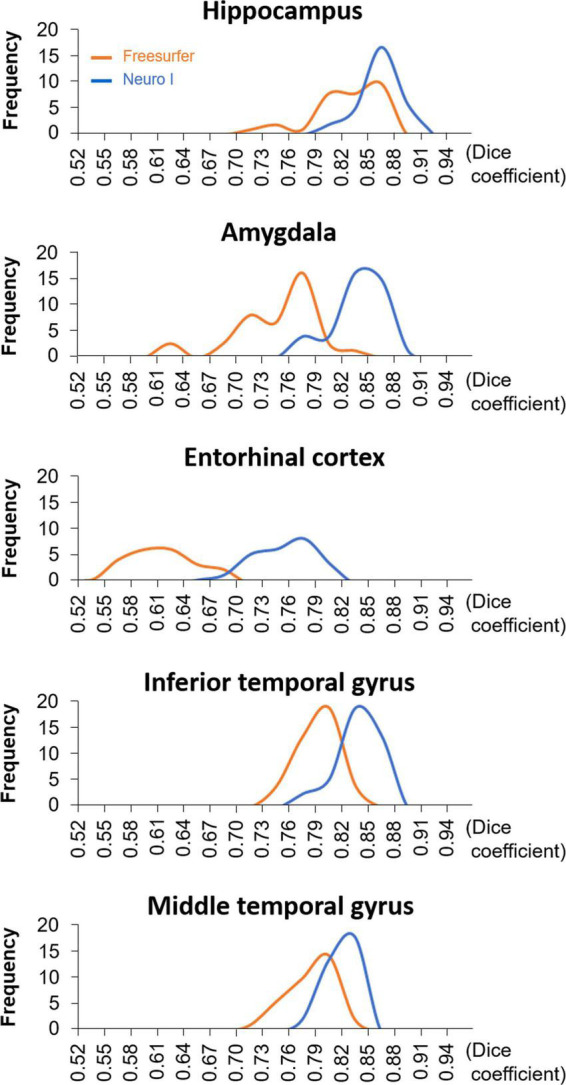
The histograms of dice coefficients obtained from Freesurfer and Neuro I.

Figure 4 shows the histograms of D values using the same data but with different segmentation approaches. The histogram of the Freesurfer results shows remarkable differences in the distribution of the D values from Neuro I, especially regarding a significant discrepancy in the amygdala and entorhinal cortex.

### 3.3. Inter-method reliability

**FIGURE 5:**
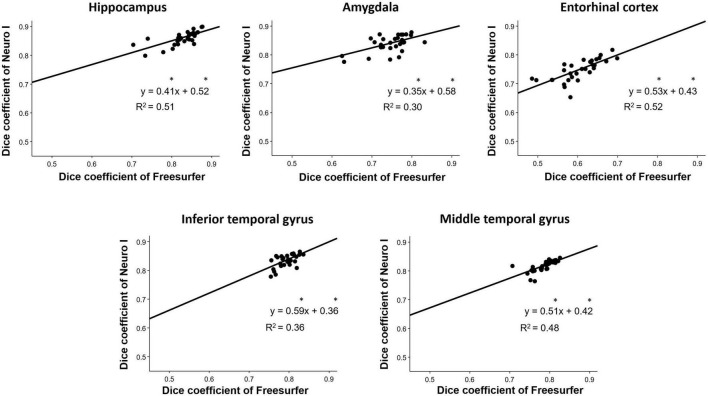
Correlation analyses for the investigation of discrepancies between dice coefficients obtained from Freesurfer and Neuro I. *Indicates that slope and intercept are significantly different from 1 and 0, respectively.

Overall D values by Freesurfer and Neuro I showed positive correlations in the hippocampus (*R*^2^ = 0.51), amygdala (*R*^2^ = 0.30), entorhinal cortex (*R*^2^ = 0.52), inferior temporal gyrus (*R*^2^ = 0.36), and middle temporal gyrus (*R*^2^ = 0.48) (Figure 5). However, surprisingly, for all ROIs, the slopes and intercepts were significantly different from 1, indicating proportional bias, and different from 0, indicating constant bias.

Regarding effect sizes, all ROIs showed that the largest effect sizes ranged from 1.07 to 3.22, especially in the amygdala and entorhinal cortex (Table 2). Also, ICCs showed significantly poor to moderate correlations between the two methods (0.498 ≤ ICC ≤ 0.688) (Table 2).

**TABLE 2:** Inter-method reliability in dice coefficients between Freesurfer and Neuro I.

Region	Effect size	ICC (95% CI)^a^
Hippocampus	1.07	0.618 (0.337–0.798)
Amygdala	2.44	0.498 (0.174–0.725)
Entorhinal cortex	3.22	0.688 (0.435–0.840)
Inferiorinferior temporal gyrus	1.95	0.599 (0.310–0.787)
Middle temporal gyrus	1.47	0.656 (0.393–0.820)

ICC, intraclass correlation coefficient; CI, confidence interval.

^a^The inter-method reliability between the two methods was analyzed by the ICC test. The *p*-values of ICC were statistically significant (all *P* < 0.001).

### 3.4. Correlation of dice coefficients with age

**FIGURE 6:**
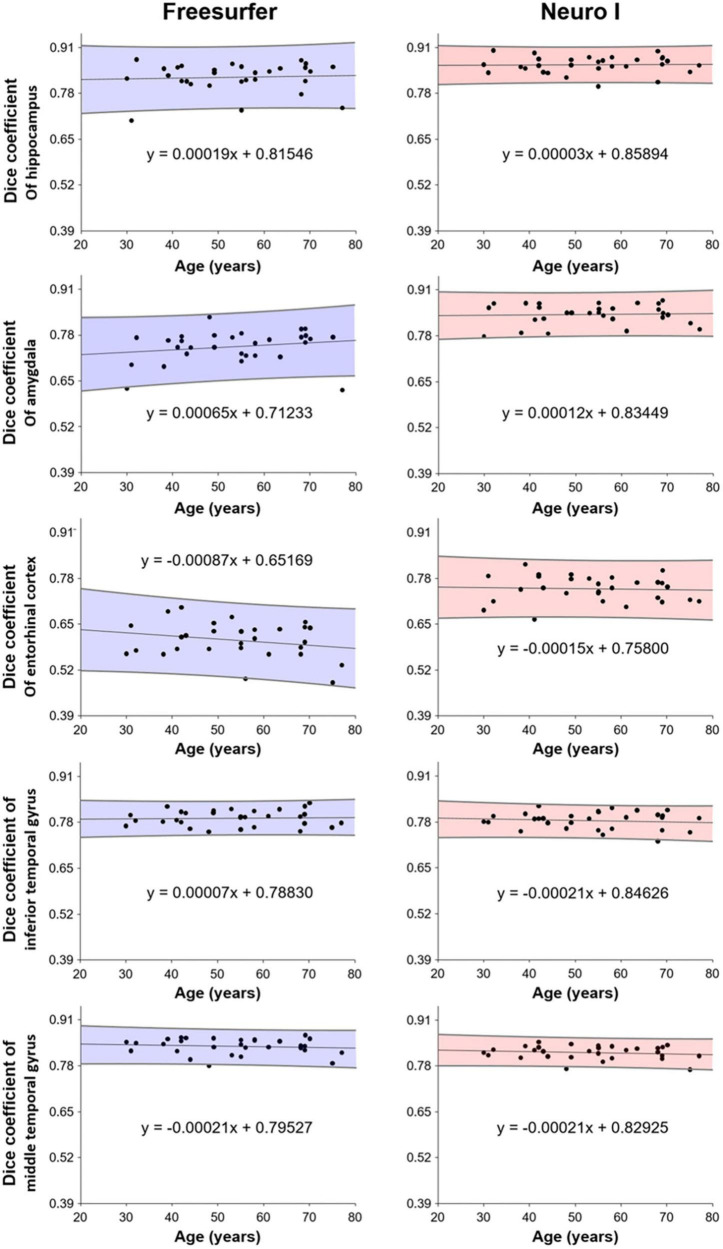
Correlation of dice coefficients (obtained from Freesurfer and Neuro I) with age.

Figure 6 shows the correlation analysis between D values and ages for each method. Our results show that segmentation strategy has a profound effect on the correlation with age. Interestingly, Neuro I revealed that D values resulted in reduced residuals when fitting data to a line of best fit, and indicated consistent values corresponding to each age, even in young and older adults.

## 4. Discussion

In this study, we evaluated brain volume measurements using Neuro I (3D CNN deep learning-based segmentation) comparing to Freesurfer (Freesurfer segmentation) in five segmented ROIs: the hippocampus, amygdala, entorhinal cortex, inferior temporal gyrus, and middle temporal gyrus. One of the disadvantages of previous segmentation strategies (e.g., Freesurfer) was the long post-processing times of 8 h (Ochs et al., 2015), while Neuro I enables processing of a whole brain MR image within 10 min. Importantly, most deep learning-based brain segmentation approaches have been oriented toward Caucasian populations; however, brain volume and shape differ between Caucasian and East Asian individuals (Kim et al., 2020). As the Neuro I deep learning model was sufficiently trained by a large cohort of East Asian individuals, we speculated that Neuro I could yield a better performance than Freesurfer.

### 4.1. Effect of different segmentation algorithms on the measurement of dice coefficients

Although Freesurfer is one of the most widely used tools in neuroimaging research for segmenting brain anatomy, several studies (Cherbuin et al., 2009; Wenger et al., 2014; Srinivasan et al., 2020) reported that the failure rate was high, resulting in exclusion of many scans, and indicating inconsistencies in segmentation. Another study revealed that the quality of volume estimation with Freesurfer may be less accurate because of over- or under-estimation in data processing. Indeed, a pediatric study (Schoemaker et al., 2016) found that, especially for volume of the hippocampus and amygdala, Freesurfer data may be inaccurate. Although our data did not include a pediatric population, when using Freesurfer, there is a slight chance that automatic segmentation may induce small errors or biases because the segmentation is not optimal. Thus, we suggest that more accurate segmentation algorithms will improve the accuracy of volume estimation and possibly increase the significance of the results.

Our hypothesis was that Neuro I would overcome some of the limitations of Freesurfer, because Neuro I has processing steps, such as analysis failure prediction, brain extraction, white matter segmentation, and analysis quality management, by applying the deep learning technique to reduce the error rates. Our results demonstrated that the Neuro I algorithm was fast and could provide high accuracy, based on brain volumes determined by T1-weighted brain MR images. In our study, Neuro I was superior to Freesurfer regarding D values in selected ROIs, indicating significant differences in mean values and IQRs compared to Freesurfer. In particular, the selected regions have been associated with biomarkers for AD diagnosis and cognitive decline (Wirth et al., 2013), and the decrease in brain volume could be explained by neuronal loss caused by amyloid deposition and neurofibrillary tangles (Goedert et al., 1991). Therefore, accurate and precise segmentation has importance in volumetry for curvature, shape, and connectivity analyses. Conversely, the underlying cause of changes in volume must be carefully considered, and the results for smaller structures should be cautiously interpreted because of potential differences due to the software used for volume measurements (Lee et al., 2021).

Comparison of histograms revealed that the D values from Freesurfer had an abnormal distribution or shape beyond the normal population range. We assumed that, using the “recon-all” command, Freesurfer might fail to process of brain images due to mismatched coordinates corresponding to a standard template or heterogeneous intensity ranges. In contrast, the model of Neuro I trained geographic patterns and image properties of brain anatomy from 776 data samples, provides a major difference from the Freesurfer algorithm.

### 4.2. Inter-method reliability

In this validation study of inter-method reliability, we found poor to moderate correlations and reliability between Freesurfer and Neuro I for the ROIs. Two studies (Fischl, 2012; Reid et al., 2017) reported that difficulty in segmenting the brain region stems from variability within its boundaries with hypointense T1 signal and a gradient of brighter intensities as one moves laterally blending with the adjacent white matter. In fact, based on visual inspection of segmentation maps, variability in the lateral boundary between the two different segmentation approaches was a contributing factor to lower inter-method reliability. Indeed, the deep learning model of Neuro I improves the quality of segmentation by producing smoother boundaries that follow the anatomic border more closely. One potential benefit of a deep learning-based brain segmentation tool is that by training over multiple samples, the model learns that jagged or stair-step boundaries are not consistent (Kaku et al., 2019). Moreover, our ROIs showed large effect sizes, which implies that the results between the two software programs were not identical.

### 4.3. Different segmentation approaches with age

Further, Neuro I (comparing to Freesurfer) showed a high success rate: segmentations in the finer anatomic regions were more consistent with ground truth segmentations, without bias with participant age. When evaluating age effects on brain volumes, this is an important finding. Participants in our study had a wide age range (30-77 years), and Neuro I showed a high level and small dispersion of D values, corresponding to age in brain regions closely associated with aging. It can be assumed that Freesurfer induces spurious age effects, which can lead to false biologic interpretations. One reason could be that Freesurfer does not use a population-based specific template (Srinivasan et al., 2020).

With high-speed data processing, which is a major advantage for Neuro I over Freesurfer, accurate deep learning-based automatic brain segmentation can screen or predict patients with cognitive impairment in clinical practice. Even though we did not display all results in detail in this paper, Neuro I can interact directly with a picture archival and communication system or Web server remotely. In addition, our final report provides the structural volumes of anatomical structures in cubic centimeters, and intracranial volumes as percentages. A normative range, relative to the East Asian standard template generated from 1,500 of healthy Koreans, is also provided for all the brain regions. Consequently, our findings may broaden the clinical feasibility of deep learning-based automatic brain segmentation, and the choice of segmentation strategy can impact the efficiency and detection capability of the volumetric analysis. Future studies are warranted to evaluate specific measures as biological markers in patients with cognitive impairment. Further, clinicians and researchers should consider the type of software used when interpreting the results of volume measurements.

### 4.4. Limitations

Our study has some limitations: first, because of the small sample size of healthy participants, there was potential for selection bias; and second, our outcomes may not be applicable to other imaging modalities, such as diffusion or perfusion MRI, or computed tomography.

## 5. Conclusion

Our Neuro I and Freesurfer were not equivalent when compared to a ground truth, especially for the segmentation of five ROIs, where Neuro I exhibited better performance. Therefore, we suggest that Neuro I is a useful alternative to assess the volume of a ROI; Neuro I can be used not only for voxel-wise analysis, but also for large-scale analysis of subcortical regions.

## Data availability statement

The original contributions presented in this study are included in the article, further inquiries can be directed to the corresponding authors.

## Ethics statement

The studies involving human participants were reviewed and approved by the Ethics Committee of Chonnam National University Hospital. Written informed consent for participation was not required for this study in accordance with the national legislation and the institutional requirements.

## Author contributions

C-MM, YYL, S-SS, and SKK designed the study. C-MM, YYL, and K-EH performed the majority of experiments. C-MM, YYL, WY, BHB, and S-HH contributed to the analysis and interpretation of results. C-MM and YYL wrote the first draft of the manuscript. S-SS and SKK have approved the final manuscript and completed the manuscript. All authors agreed with the content of the manuscript.
